# Uncovering jaw-specific radiographic differences in medication related osteonecrosis of the jaws (MRONJ): a case-control study

**DOI:** 10.1007/s00784-026-06837-4

**Published:** 2026-03-27

**Authors:** Daya Masri, Gavriel Chaushu, Eli Rosenfeld, Daniel Muchnik, Shaked Adut, Gal Avishai, Omar Ghanaiem

**Affiliations:** 1 Department of Oral and Maxillofacial Surgery, The Maurice and Gabriela Goldschleger School of Dental Medicine, Tel Aviv University, Tel Aviv, 69978 Israel; 2 Department of Oral and Maxillofacial Surgery, Rabin Medical Center – Belinson Hospital, Petah Tikva, 49414 Israel; 3 Department of Oral and Maxillofacial Surgery, Rabin Medical Center, Petah Tikva, Israel

**Keywords:** MRONJ, Radiographic features, Maxilla versus mandible, Sclerosis, Osteolysis, Sequestration, Periosteal reaction

## Abstract

**Objective:**

To compare the radiographic manifestations of medication-related osteonecrosis of the jaw (MRONJ) between the maxilla and the mandible using a matched case–control design, and to identify jaw-specific imaging features that may support early diagnosis.

**Materials and methods:**

A retrospective analysis was conducted on 109 MRONJ patients treated between 2013 and 2024 at a tertiary care center. A matched cohort of 45 patients (15 maxillary, 30 mandibular) was created based on age (± 2 years), gender, and underlying condition (osteoporosis vs. malignancy). Demographic, clinical, and radiographic data using cone-beam computed tomography (CBCT) or multidetector computed tomography (MDCT), were extracted and analyzed. Radiographic features evaluated included osteolysis, sclerosis, periosteal bone formation, and sequestration.

**Results:**

Demographic and clinical variables were similar between the groups. Radiographically, osteolysis was significantly more common in maxillary MRONJ (93.3% vs. 63%, *P* = 0.0319), while the mandible showed higher frequencies of periosteal reaction (50% vs. 6.6%, *P* = 0.0042) and sequestration (80% vs. 33.3%, *p* = 0.0021). Sclerosis was more common in mandibular cases (90% vs. 73.3%) but did not reach statistical significance (*P* = 0.1458). No significant differences were observed in MRONJ staging or comorbidities (*P* > 0.05 respectively).

**Conclusion:**

Maxillary and mandibular MRONJ demonstrate distinct radiographic patterns, likely reflecting anatomical and vascular differences. The predominance of osteolysis in the maxilla, which often presents with less specific symptoms, may contribute to delayed diagnosis. Recognizing these jaw-specific imaging features can improve diagnostic accuracy and promote earlier intervention, particularly for maxillary MRONJ.

**Clinical relevance:**

By uniquely applying a matched case–control design, this study uncovers a jaw-specific radiographic signatures of MRONJ—highlighting the overlooked dominance of osteolysis in the maxilla and the aggressive periosteal and sequestration changes in the mandible—offering a powerful diagnostic lens for earlier, site-specific intervention.

## Introduction

Medication-related osteonecrosis of the jaw (MRONJ) is a severe adverse effect of antiresorptive and antiangiogenic therapy, commonly used in osteoporotic or cancer patients [1, 2]. It is diagnosed based on persistent exposed bone for over eight weeks in the absence of prior radiation or metastasis, and is staged according to lesion severity, ranging from asymptomatic bone exposure (Stage 1) to extensive necrosis involving adjacent structures or complications like fractures (Stage 3) [1–6]. Though MRONJ is rare in osteoporotic patients, it occurs significantly more often in cancer patients due to higher drug doses and frequencies [2, 7–9]. Affected individuals often suffer substantial declines in quality of life due to chronic pain, infections, and functional impairments, especially among elderly or medically compromised populations [1, 10–13]. Diagnosis is usually established at advanced stages, primarily due to limited awareness among dental professionals, underscoring the need for improved recognition, early detection, and education regarding MRONJ’s clinical and radiographic features [14–16].

MRONJ lesions present with a variety of radiographic features, from sclerosis to sequestration, osteolysis, periosteal new bone formation, thickening of the lamina dura and periodontal ligament loss, that reflect the pathophysiologic event of the bone necrosis, impaired remodeling, and secondary infection [2, 17] (Figs. 2, 3 and 4). Sclerosis, one of the earliest signs of MRONJ lesion, expressed as an area of increased bone density, reflecting suppressed bone turnover caused by antiresorptive therapy and chronic inflammation, whereas lysis represents bone destruction resulting from progressive necrosis and secondary infection [18, 19] (Fig. 2). Sequestration can occur in advanced lesions when necrotic bone becomes isolated from the adjacent living bone after the loss of its blood supply[20] (Figs. 2, 3 and 4). Besides, periosteal reaction showing a non-specific bone healing attempt at the cortical margin [21] (Fig. 2). Recognizing these radiographic features along with information regarding the patient’s health history, enables clinicians to diagnose and manage MRONJ promptly in an appropriate time.

Epidemiologically, MRONJ is far more common in the mandible than in the maxilla, with mandibular cases occurring about three times as often as maxillary cases. The difference is mainly contributed to embryological, anatomical and physiological reasons: the compromised vascularity of the mandible and its compact bone make it more susceptible to necrosis and impaired healing, while the more profuse vascularity and porous bone of the maxilla promote higher resistance and healing [22, 23].

Diagnosis of maxillary MRONJ is often delayed compared to mandibular cases. This is largely due to the fact that lesions in the maxilla tend to present with vague or nonspecific symptoms, which are frequently misinterpreted as sinusitis or other Sino-nasal disorders because of their close anatomical relationship to the maxillary sinus. Maxillary MRONJ therefore goes unnoticed or is misdiagnosed, resulting in postponed identification and treatment [24, 25]. In contrast, mandibular MRONJ typically presents with more recognizable signs, such as an intra-oral exposed bone or an extra-oral fistula near the jaw, making it easier for dental and medical professionals to identify [26].

Due to its high healing capacity, maxilla shows lower incidence and generally milder manifestation of MRONJ, even in long-standing disease. This is reflected in both the smaller number of maxillary MRONJ cases and the typically lower disease stage when the maxilla is affected. For example, Okuyama et al. evaluated 54 maxillary cases of MRONJ and concluded that 37 cases (68%) were either stage 1 or 2, while only 17 cases (32%) were stage 3, highlighting the tendency for less severe disease in the maxilla [25]. We would assume that the ability of the maxilla to regenerate much better would be one of the reasons for its resistance and preservation of less severe stages.

Because MRONJ is more commonly found in the mandible, the majority of published studies and case series have been concerned with its diagnosis and treatment in this site, with general lack of literature on MRONJ imaging and limited comparative data on the radiographic presentation of maxillary versus mandibular lesions [22, 23, 27]. Yet, effective management of maxillary lesions is particularly crucial, as these can progress more extensively and lead to severe complications such as oroantral communication (OAC), oroantral fistula (OAF), sinusitis, nasal septal abscess, orbital cellulitis, skull base necrosis, and even brain abscess [11, 23].

This study aims to identify and describe any distinct radiographic features of MRONJ in the maxilla versus the mandible, with the goal of improving diagnostic accuracy and predictability. By focusing on the specific radiographic characteristics of maxillary MRONJ, often overlooked due to its subtle presentation, this study seeks to addresses a critical gap in the current literature. A greater understanding of jaw-specific imaging features in MRONJ has the potential to significantly reduce delays in diagnosis, improve treatment outcomes, and ultimately mitigate severe complications associated with maxillary involvement. In turn, better diagnostics can significantly improve quality of life for patients. By providing clinicians with improved and earlier diagnosis, this study could lead to effective interventions.

## Materials and methods

### Study cohort

This retrospective cohort study was approved by the Institutional Review Board of Rabin-Beilinson Medical Center (Approval No. RMC-0160-23). The study included all patients diagnosed with medication-related osteonecrosis of the jaw (MRONJ) who were referred to the Department of Oral and Maxillofacial Surgery at Rabin Medical Center, Beilinson Campus (Petah-Tikva, Israel), a tertiary referral center, from 2013 to 2024. MRONJ was diagnosed according to the clinical and radiographic criteria outlined in the 2022 guidelines of the American Association of Oral and Maxillofacial Surgeons (AAOMS), and was histopathologically confirmed in some cases. Clinical data were obtained through a review of electronic medical records.

### Cohort selection

Study cohort patients were selected from a larger institutional cohort of 210 individuals diagnosed with MRONJ between 2013 and 2024. Within this main cohort, only 23 patients presented with maxillary MRONJ. A case–control database was constructed by matching maxillary MRONJ cases to mandibular MRONJ controls to reduce confounding and intergroup variability.

Matching was performed using a 1:2 ratio (case: control). For each maxillary MRONJ patient, two mandibular MRONJ patients were selected based on the following predefined criteria: age group (± 2 years), gender, and underlying disease indication for antiresorptive therapy (oncologic vs. osteoporotic).

Of the 23 maxillary MRONJ patients, 15 could be successfully matched to two appropriate mandibular MRONJ controls according to all matching criteria. The remaining eight maxillary MRONJ patients were excluded from the matched analysis because no suitable mandibular MRONJ controls meeting all matching requirements were available, primarily due to discrepancies in age range and/or underlying disease indication.

The final matched cohort therefore consisted of 45 patients, including 15 maxillary MRONJ cases and 30 matched mandibular MRONJ controls, which formed the basis for subsequent comparative analyses.

**Fig. 1:**
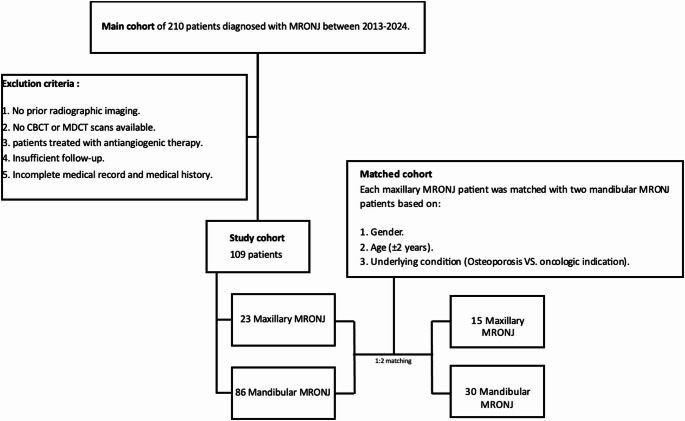
Cohort selection

The composition of the main cohort, the selection of the study cohort, and the final matched cohort are illustrated in (Fig. 1). Chart reviews were independently conducted by two senior maxillofacial surgeons (G.C. and D.M.).

### Inclusion criteria

1. Patients diagnosed with MRONJ who attended the Oral and Maxillofacial Surgery (OMS) outpatient clinic between 2013 and 2024.
2. Patients with a minimum follow-up period of at least one year.
3. Patients with documented pre-treatment radiographic imaging using cone-beam computed tomography (CBCT) or multi-detector computed tomography (MDCT).

### Exclusion criteria

1. Patients who attended the outpatient clinic without prior radiographic imaging.
2. Insufficient clinical and radiographic (CBCT or MDCT) follow-up.
3. Oncologic patients treated with antiangiogenic therapy.
4. Incomplete medical record and medical history.
5. Patients with metastatic disease to the jaws.

### Data collection

Data were extracted from the hospital’s electronic medical records system using a formatted form including; *Demographic and Clinical Data -*Age (years), Gender (Male, Female), Underlying condition (oncologic vs. osteoporotic indication) and Relevant medical comorbidities: hypothyroidism, diabetes mellitus, anemia, smoking status, hypertension, hyperlipidemia and steroid exposure. *And MRONJ-Specific Data-*Location of the lesion (maxilla or mandible). The region of involvement was further classified based on an anterior–posterior anatomical definition: the anterior region included the area between the canines, while the posterior region comprised the premolar and molar areas. Lesions involving the ramus of the mandible or the maxillary sinus region were not excluded; instead, they were categorized according to this anterior–posterior definition based on their primary site of involvement. Staging *(1–3)*,* Type* of antiresorptive medication (bisphosphonates vs. Denosumab), Duration of drug exposure (in years) *and* radiographic features *(*sclerosis, sequestration, osteolysis, periosteal new bone formation).

CBCT images were obtained using the Kavo OP 3D (Kavo Dental, Germany), whereas MDCT images were collected with the Siemens Somatom Force (Siemens Healthineers, Germany). Both imaging modalities were operated under the following parameters: KVp 90–120, mA 5–25, voxel size 0.25 mm, and slice thickness of 1 mm. The acquired images were subsequently processed and analyzed utilizing the Philips PACS system.

Two senior maxillofacial surgeons (G.C. and D.M.) independently reviewed the radiographic images and determined the presence or absence of each radiographic feature. In cases where the initial reviewers disagreed, a third senior maxillofacial surgeon (E.R.) performed a final inspection to resolve the discrepancy and establish a consensus diagnosis. The radiographic evaluation performed based on the following operational definitions;

- Osteolysis was defined as focal or diffuse loss of normal bone density relative to adjacent uninvolved bone, characterized by ill-defined or geographic radiolucency with disruption or thinning of the normal trabecular architecture; cortical involvement was considered present when endosteal scalloping of the cortex was present or when focal cortical interruption was identified.
- Sclerosis, defined as an abnormal increase in bone density relative to adjacent bone, manifesting as increased trabecular thickness with reduced inter-trabecular spaces or homogeneous medullary opacity replacing the normal trabecular pattern; compared with adjacent or contralateral normal bone, and only sclerosis contiguous with areas of suspected infection was considered MRONJ related.
- Periosteal reaction was defined as new bone formation along the external cortical surface, appearing as linear, lamellated, or irregular mineralized densities paralleling the cortex and separated by a radiolucent line, extending longitudinally and clearly distinguishable from adjacent and contralateral normal bone tissue.
- Sequestration was defined as a devitalized bone fragment appearing as a well-defined radiodense focus within an area of osteolysis, with density equal to or greater than adjacent cortical bone, surrounded partially or completely by a radiolucent margin, and lacking continuity with surrounding trabecular or cortical structures.

It is important to note that radiographic features such as lamina dura thickening or periodontal ligament space destruction were not evaluated in this study since these are not optimally appreciated on CT scans.

### Statistical analysis

All statistical analyses were conducted using SPSS version 30. Descriptive statistics were applied to summarize demographic and clinical variables. Continuous variables were reported as mean ± standard deviation (SD) and range when appropriate. Categorical variables were expressed as frequencies and percentages.

Normality of continuous variables was assessed using the Shapiro–Wilk test. Group comparisons of continuous variables were done using independent samples t-test or Mann–Whitney U test, depending on the distribution. Categorical variables were compared using the chi-square test or Fisher’s exact test, as appropriate. Comparisons between the maxilla and mandible groups within the matched cohort (1:2 matching based on age [± 2 years], sex, and underlying condition—malignancy or osteoporosis) were performed using the chi-square test to assess differences in categorical variables. A statistical significance was defined as a p-value < 0.05. Finally, inter-rater reliability was calculated using Cohens Kappa test.

## Results

A total of 45 patients were included in the matched case-control study: 15 with maxillary MRONJ and 30 with mandibular MRONJ. MRONJ diagnosis was established based on clinical examination and medical history, with histopathological confirmation available for 10 of 15 maxillary lesions (66.7%) and 20 of 30 mandibular lesions (66.7%). The average age was similar between the maxillary (70 ± 9.3 years, range 54–90) and mandibular (70 ± 8.6 years, range 53–91) groups (*P* = 0.943). Gender and underlying conditions (osteoporosis vs. oncologic indications) were also evenly distributed between the groups (*P* = 1.0 for both). No statistically significant differences were found in the prevalence of common comorbidities such as hypothyroidism, diabetes mellitus, steroid use, anemia, or smoking (*P* > 0.05 for all). The distribution of antiresorptive therapy type (bisphosphonates vs. denosumab) was also not statistically different between the maxillary and mandibular groups (*P* = 0.1967 for both). The average duration of antiresorptive medication use was similar in both groups (maxilla: 3.4 ± 2.1 years; mandible: 3.5 ± 2.8 years; *P* = 0.484). Similarly, there was no statistically significant difference in the distribution of MRONJ stages between the maxillary and mandibular cases (*P* > 0.05 for all stages). Lesion location (anterior vs. posterior) was also not significantly different between the two groups (*P* = 0.1458) (Table 1).

**Table 1:** Comparison of Demographic, Clinical Characteristics Between Maxillary and Mandibular MRONJ Patients (Matched Cohort)

Average age ± SD (range)	Maxilla (*n* = 15)	Mandible (*n* = 30)	*P*-value	95% Confidence Interval
	70 ± 9.3 (54–90)	70 ± 8.6 (53–91)	0.943	-
Gender				
Male (n,%)	5 (33.3%)	10 (33.3%)	1	-
Female (n, %)	10 (66.6%)	20 (66.6%)		-
Underlying condition				
Osteoporosis (n,%)	5 (33.3%)	10 (33.3%)	1	[−0.272, 0.272]
Oncologic n (%)	10 (66.6%)	20 (66.6%)		[−0.272, 0.272]
Predisposing factors				
Hypothyroidism (%)	3 (20.0%)	2 (6.0%)	0.17971	[−0.075, 0.342]
Diabetes Miletus (%)	3 (20.0%)	6 (20.0%)	1	[−0.204, 0.204]
Steroid use (%)	3 (20.0%)	6 (20.0%)	1	[−0.204, 0.204]
Anemia (%)	2 (13.3%)	3 (10.0%)	0.7373	[−0.143, 0.210]
Smoking (%)	5 (33.3%)	7 (23.0%)	0.4745	[−0.132, 0.331]
Antiresorptive therapy				
Bisphosphonates (%)	7 (46.6%)	20 (66.6%)	0.1967	[−0.449, 0.049]
Denosumab (%)	8 (53.3%)	10 (33.3%)		[−0.449, 0.049]
Duration of intake in years (Average ± SD, Range)	3.4 ± 2.1 (0.5–7.5)	3.5 ± 2.8 (0.5–15)	0.4843	-
MRONJ Stage				
Stage 1	4 (26.6%)	5 (16.6%)	0.4291	[−0.091, 0.291]
Stage 2	6 (40.0%)	14 (46.6%)	0.6713	[−0.302, 0.170]
Stage 3	5 (33.3%)	11 (36.6%)	0.8257	[−0.272, 0.205]
Region of involvement				
Anterior region (canine – canine)	4 (26.6%)	3 (10.0%)	0.1458	[−0.026, 0.359]
Posterior region (premolar – molar)	11 (73.3%)	27 (90.0%)		[−0.438, 0.104]

However, significant differences were observed in radiographic findings:

- **Osteolysis (Lytic Lesions)**: Significantly more common in maxillary MRONJ (93.3%) than in mandibular MRONJ (63.0%) (*P* = 0.0319).
- **Periosteal Bone Formation**: Significantly more frequent in mandibular MRONJ (50.0%) compared to maxillary MRONJ (6.6%) (*P* = 0.0042).
- **Sequestration**: Significantly more common in mandibular MRONJ (80.0%) than in maxillary MRONJ (33.3%) (*P* = 0.0021).
- **Sclerosis**: While more common in mandibular cases (90.0% vs. 73.3%), this difference was not statistically significant (*P* = 0.1458).

**Fig. 2:**
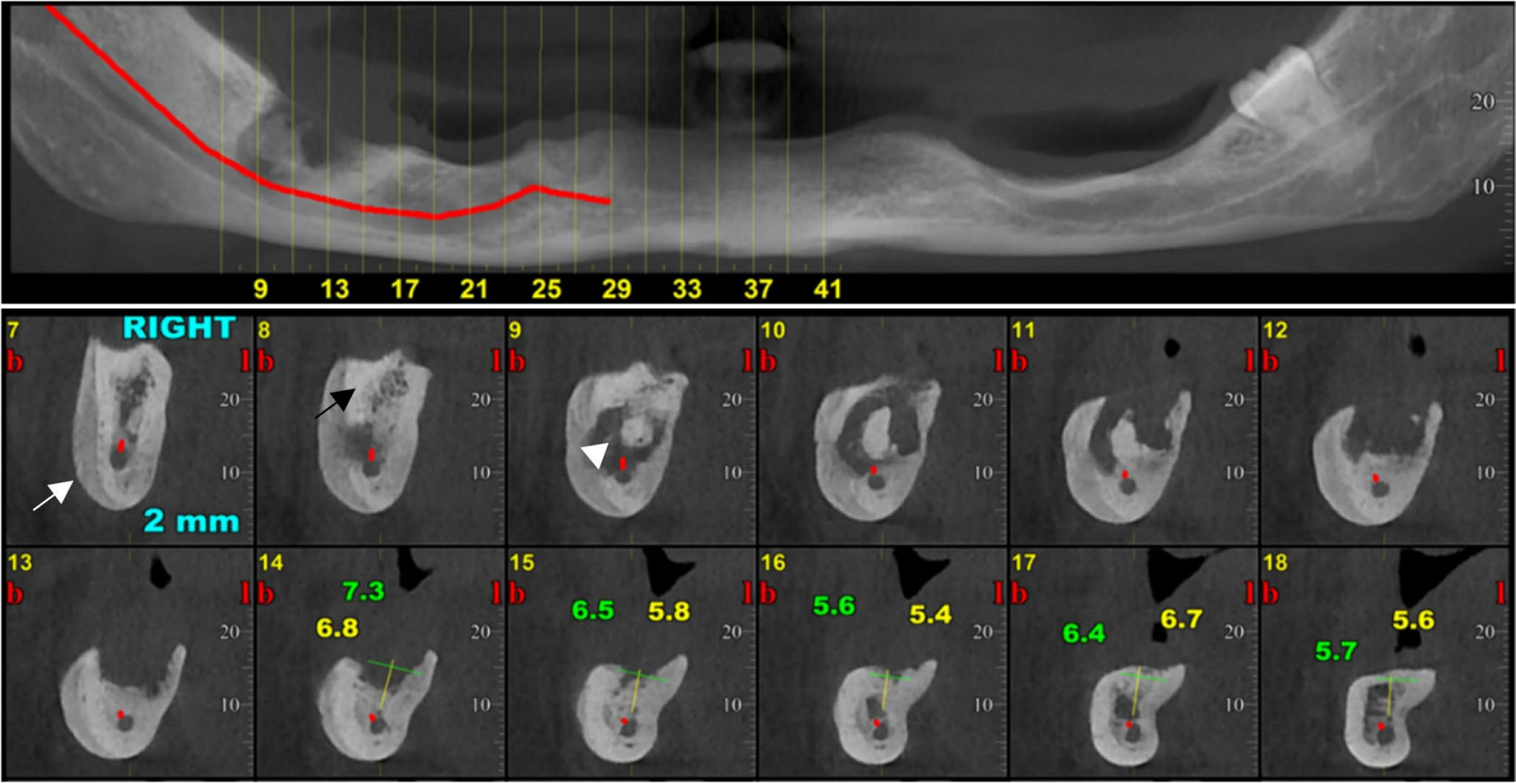
Right mandibular MRONJ lesion demonstrating periosteal bone formation (white arrow), sequestration (white arrow head), and sclerosis (black arrow)

**Fig. 3:**
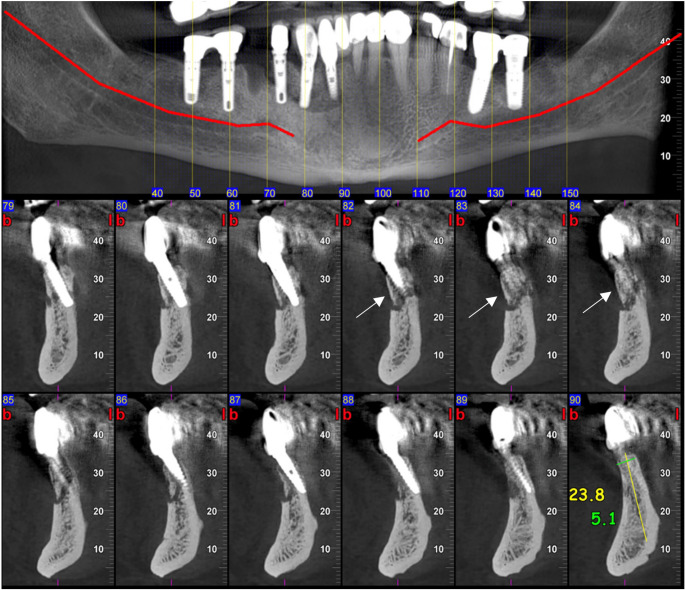
Right mandibular peri-implant MRONJ lesion demonstrating sequestration (white arrow)

**Fig. 4:**
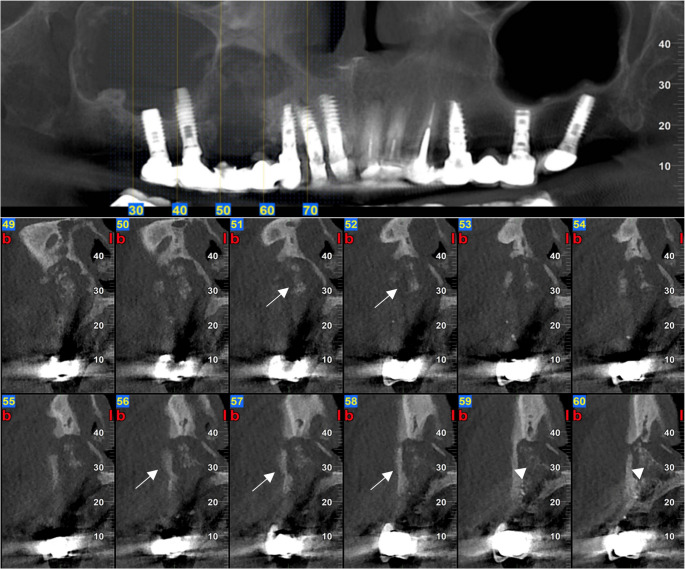
Right maxillary MRONJ lesion involving the righ maxillary sinus demonstrating sequestration (white arrow) and lysis (white arrow head)

Radiographic findings are summarized in Table 2 and illustrated in Figs. 2, 3 and 4.

**Table 2:** Comparison of Radiographic Characteristics Between Maxillary and Mandibular MRONJ Patients (Matched Cohort)

Lysis	Maxilla (*n* = 15)	Mandible (*n* = 30)	*P*-value	95% Confidence Interval
	14 (93.3%)	19 (63.0%)	0.0319	[0.032, 0.575]
Sclerosis	11 (73.3%)	27 (90.0%)	0.1458	[−0.438, 0.104]
Periosteal bone formation	1 (6.6%)	15 (50.0%)	0.0042	[−0.692, −0.175]
Sequestration	5 (33.3%)	24 (80.0%)	0.0020	[−0.707, −0.226]

Statistically significant P-values (*P* < 0.05)

### Inter-observer agreement and reliability

the present study, involving 45 patients, aimed to identify four specific radiographic features through assessments by two maxillofacial surgeons. The results demonstrated a high level of agreement between the observers (Table 3). For osteolysis, they concurred in 44 out of 45 cases, while the consensus for sclerosis was similarly high at 44 cases and total agreement for sequestration, identifying it consistently in all 45 cases. However, for periosteal reaction, agreement was reached in 42 cases, indicating some variability in interpretation. Consequently, a third observer was required in five instances to resolve discrepancies and ensure accurate diagnoses.

Statistically significant P-values (*P* < 0.05), SD= Standard deviation, n=Number.

**Table 3:** Inter-observer agreement and reliability

Radiographic Feature	Total Cases	Agreement Count	Disagreement Count	Observed Agreement	Kappa Score	Interpretation
Sequestration	45	45	0	100%	1	Perfect Agreement
Lysis	45	44	1	97.8%	0.95	Almost Perfect
Sclerosis	45	44	1	97.8%	0.95	Almost Perfect
Periosteal Bone Formation	45	42	3	93.3%	0.83	Almost Perfect

## Discussion

To our knowledge, this is the first systematic comparison of the radiographic appearance of MRONJ in the maxilla versus the mandible in matched case control cohort extracted from a larger cohort. This made it possible for us to reduce potential confounders and enhance the generalizability of our findings. Our results show clear anatomical and radiographic distinctions between the two mandibles and maxillae, offering insights that may aid in diagnosis, risk stratification, and potentially prognosis.

Although our study was based solely on CBCT and MDCT imaging, it is important to acknowledge the role of other imaging modalities, particularly MRI, in the detection of MRONJ. MRI is highly sensitive for early disease, as it can identify bone marrow and soft tissue changes before clinical or radiographic abnormalities become evident, making it especially valuable in non-osteolytic cases [28, 29]. However, MRI is less effective than CT/CBCT for evaluating cortical bone changes including osteolysis and sequestration [30], and its routine use may be limited by cost, availability, contraindications, longer acquisition times, and potential interpretative challenges.

Demographic and clinical characteristics - age, gender, underlying conditions, and most comorbidities were largely comparable between maxillary and mandibular MRONJ patients (Table 1). This comparability supports the internal validity of our findings and allows us to attribute the observed radiographic differences primarily to anatomical and biological factors rather than confounding variables.

The findings confirm that radiographic manifestations of MRONJ are typically more pronounced in the mandible. Specifically, three of four radiographic features (sequestration, periosteal bone formation and sclerosis) were significantly more prevalent in mandibular MRONJ, whereas osteolysis was the most prominent feature in maxillary cases (Table 2), consistent with previous observations [31] This differential radiographic presentation can assist with the clinical diagnostic challenge of maxillary MRONJ, which often presents with subtler radiological characteristics. Once solely osteolysis is observed in the maxilla, the possible diagnosis of MRONJ should alert the clinician even without additional radiographic signs. Additional evidence in favor of MRONJ diagnosis should be derived from the medical history and clinical examination.

Anatomical and vascular distinctions between in the jaws offer a good explanation for these differences. The mandible relies mainly on the inferior alveolar artery for endosteal perfusion with minor periosteal contribution; while vascular contributions from adjacent arteries are present, the overall capacity for collateral circulation is relatively limited compared with the maxilla [2, 32, 33]. Furthermore, its compact cortical bone further restricts vascular penetration [34]. On the contrary, the maxilla receives a more profuse, redundant blood supply from multiple branches of the maxillary artery including the posterior, middle, and anterior superior alveolar arteries, as well as contributions from the greater palatine and sphenopalatine artery [35]. In addition, the maxilla possesses a more porous and vascular bony anatomy [34, 36, 37]. These can be the explanation for greater susceptibility of the mandible for ischemic complications and the typical radiographic appearance of MRONJ observed in the present study on one hand and makes the maxilla more susceptible to osteolysis on the other hand.

Sequestration, defined as infection-induced avascular necrosis [38], was much more frequent in the mandible (Table 2). This is consistent with the mandible’s compromised vascularity and diminished capacity for healing [32, 34]. Similarly, periosteal bone formation—a reactive osteogenic process in response to chronic infection [21, 39, 40]—was also more common in the mandible (Table 2). Anatomically the cortical bone of the mandible is clearly evident while the maxilla demonstrates negligible cortex. This has been supported by previous studies showing that the periosteum’s regenerative potential varies by anatomical location, with mandibular periosteal cells demonstrating a more robust osteogenic response than those in the maxilla. Yuka et al. further outlines a significant association between mandibular involvement and periosteal reaction and proposed it as a potential prognostic indicator [41].

While sclerosis was seen in both jaws, it did not differ significantly between groups. This finding is consistent with earlier reports by Ghanaiem et al., who found increased sclerosis in osteoporotic patients [31], and Shin et al. [42], who found no difference between oncologic and osteoporotic groups. collectively, these findings suggest that sclerosis may be a general feature (non-specific) of chronic bone turnover in MRONJ, independent of jaw location.

Conversely, osteolysis was significantly more frequent in maxillary MRONJ. This is likely due to the abundant vascularity of the maxilla, permitting more diffuse inflammatory and osteolytic response to infection [35]. Interestingly, Yuka et al. also observed that an absence of osteolysis in mandibular MRONJ was associated with poorer prognosis, raising the possibility that the presence of osteolysis may indicate a more effective immune response or earlier disease phase [43]. The higher rate of osteolysis in maxillary MRONJ, in addition to its overall lower incidence and potentially less aggressive radiographic pattern, may help explain the more indolent clinical course that is often observed in such patients.

## Limitations

A relatively small number of maxillary MRONJ cases and a retrospective design; the inherently subjective nature of radiographic assessment, which we sought to mitigate by having two senior maxillofacial surgeons (G.C. and D.M.) independently evaluate images with a third senior surgeon (E.R.) resolving disagreements; potential selection bias because only patients with available CT scans—often those with more severe disease—were included; and intrinsic limits of imaging modalities like CBCT/MDCT, which may miss early or subtle MRONJ lesions in the maxilla due to overlapping anatomy and limited soft-tissue contrast, obscuring subtle marrow changes, early periosteal reactions, or small osteolytic areas. Moreover, because MRI is considered the imaging modality of choice for osteomyelitis and MRONJ, our reliance on CT represents an additional limitation; Future prospective, multicenter studies, incorporation of advanced imaging such as MRI, and histological analysis are needed to confirm these results and better establish the diagnostic and prognostic value of radiographic features across jaw locations.

## Conclusions

The radiographic characteristics of MRONJ is extremely variable across maxilla and mandible, reflecting underlying anatomical and physiological differences. The mandible demonstrates more quickly progressing, aggressive destructive changes in the form of sequestration and periosteal reaction, while the maxilla displays slowly progressing, less caustic lytic lesions. It is important to recognize these differences in order to prevent delay in diagnosis, increase accuracy and guide early intervention.

## Data Availability

No datasets were generated or analysed during the current study.
